# The oscillation-outbreaks characteristic of the COVID-19 pandemic

**DOI:** 10.1093/nsr/nwab100

**Published:** 2021-06-08

**Authors:** Jianping Huang, Xiaoyue Liu, Li Zhang, Yingjie Zhao, Danfeng Wang, Jinfeng Gao, Xinbo Lian, Chuwei Liu

**Affiliations:** Collaborative Innovation Center for Western Ecological Safety (CIWES), Lanzhou University, China; Collaborative Innovation Center for Western Ecological Safety (CIWES), Lanzhou University, China; Collaborative Innovation Center for Western Ecological Safety (CIWES), Lanzhou University, China; Collaborative Innovation Center for Western Ecological Safety (CIWES), Lanzhou University, China; Collaborative Innovation Center for Western Ecological Safety (CIWES), Lanzhou University, China; Collaborative Innovation Center for Western Ecological Safety (CIWES), Lanzhou University, China; Collaborative Innovation Center for Western Ecological Safety (CIWES), Lanzhou University, China; Collaborative Innovation Center for Western Ecological Safety (CIWES), Lanzhou University, China

## Abstract

The evolution of the COVID-19 pandemic features the alternation of oscillations and abrupt rises. The oscillations are attributable to weekly and seasonal modulations, while abrupt rises are stimulated by mass gatherings.

SARS-CoV-2 has been circulating in the human population for more than a year and has caused over 150 million cases globally as of 1 May 2021. Although a lot of regions have relied on measures such as social distancing, contact tracing and quarantine to slow its spread [1], multidisciplinary researchers are actively engaged in understanding the dynamic of its transmission. The accurate prediction of the COVID-19 pandemic is foundational to guiding public health policy-making and alleviating socio-economic consequences [2,3]. Since the beginning of the outbreak, numerous studies have used diverse techniques to assess the disease’s transmission dynamics and predict its future course [2–4]. However, these modeling results have shown a wide range of variations. Fundamental improvements to the prediction require a deeper and wider understanding of the transmission dynamics under both human interventions and environmental influence. Here we provide an in-depth exploration of the periodicity and mutability in the evolutionary history of the COVID-19 pandemic and investigate the principle mechanisms behind them based on statistical and dynamical models.

The transmission of SARS-CoV-2 is regulated by various processes on multiple timescales. Isolating these processes on different timescales can help to identify the major inducements of the COVID-19 pandemic. We used the Ensemble Empirical Mode Decomposition (EEMD) method (Supplementary Section 1) to separately decompose the time series data of daily confirmed cases and deaths in the northern and southern hemispheres (NH and SH). The time series data consist of the oscillations over weekly and seasonal timescales and the long-term trend that drives the increase in COVID-19 cases (Fig. 1b). The weekly oscillations for both hemispheres exhibit negative signals for confirmed and death cases during the weekend, while positive signals are exhibited in the middle of the week (Wednesday, Thursday and Friday, see Fig. S3). This can be explained by the distinct differences in human behavior patterns between weekdays and weekends, which may lead to a weekly cycle of contact rate, contributing to higher (lower) infection possibility during weekdays (weekends). Although this weekly pattern is consistent with the observed weekly oscillation of COVID-19 daily cases, the observed oscillation is mainly attributable to the reporting bias, with higher rates of reporting during certain days of the week [5]. A higher mortality reported during weekdays further supports this point since the weekly behavior pattern is unlikely to cause a higher mortality during weekdays (Fig. S3).

**Figure 1.**
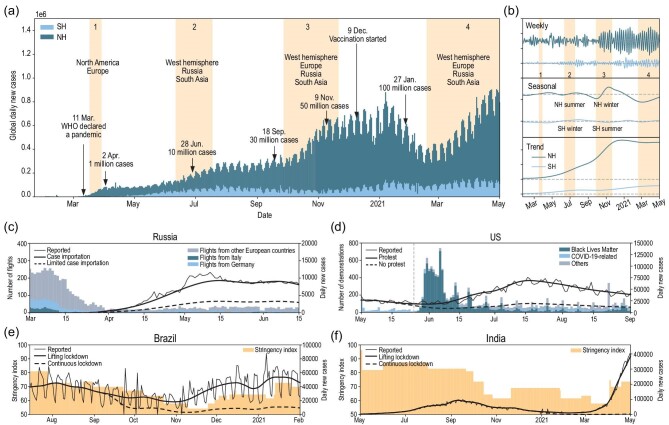
The evolution of the COVID-19 pandemic. (a) Global daily new cases, with deep (light) blue denoting the cases in the northern (southern) hemisphere. (b) The weekly, seasonal and trend components decomposed by the EEMD method. (c)–(f) show the scenario simulations in (c) Russia, (d) US, (e) Brazil and (f) India. The thin black lines in (c)–(f) denote the reported daily new cases in each country, while the thick solid and dashed lines denote the simulation in two different scenarios.

Seasonal modulation is another major factor that influences the dynamics of COVID-19 transmission. Using the EEMD method, decomposed oscillations on the seasonal time scale indicate higher infectivity and mortality in colder climates for both hemispheres, as shown in Fig. 1b (for decomposition of death cases please refer to Figs S2 and S3). This result is consistent with both epidemiological and laboratory studies [6]. Seasonal variations in meteorological and environmental factors can affect COVID-19 transmission via their influence on virus stability, host immunity and human behavior. However, the EEMD decomposition shows that the seasonal oscillations with limited amplitude are not able to reverse the long-term growing trend of the cases (Fig. 1b). Therefore, beneficial climate conditions (e.g. onset of higher temperatures during the warm seasons) should not be used as a criterion with regard to deciding whether or not to relax control measures [7,8].

The time series data of COVID-19 exhibit cyclical behavior due to seasonal and weekly modulation, while its evolution is also regulated by some rapid growth periods (abbreviated as outbreaks hereafter). These abrupt shifts could be attributable to changes in governmental response and public adherence, as well as the unexpected natural and socio-economic crisis. In either case, these incidents result in a higher risk of mass gathering, which has directly led to super-spreading events and the subsequent COVID-19 disaster. An anomaly detection algorithm (Supplementary Section 2) has identified four major outbreaks along the COVID-19 time series data, which are shaded in Fig. 1a. For each outbreak, we separately selected a hotspot region with a dominant contribution (Russia, US, Brazil and India) and attempted to provide causal explanations of these outbreaks based on the second version of the Global Prediction System for COVID-19 Pandemic (GPCP, v2, details in the Supplementary Data) [4].

Russia is among the four countries with the highest number of confirmed COVID-19 cases as of May 2020. However, during the first outbreak, the initial rise of cases in Russia happened later than many of the neighboring countries. This is possibly due to the effective implementation of proactive non-pharmaceutical interventions (NPIs), which limited the virus import from Asian countries. Unfortunately, the Russian authorities did not react quickly enough to prevent case importation from European countries, in which local transmissions had already occurred until mid-March 2020. As of 15 March, 74.2% of the inbound flights from other European countries were still operating (Fig. 1b). A recent genomic study has shown that most of the sampled sequences from Russia in the early stage are nested within other European subclades, which indicates multiple introductions of the virus from Europe [9]. Our simulation indicated that if travel restrictions were implemented five days earlier, 64.1% of the cases could have been avoided as of 20 May 2020, as shown in Fig. 1c and Supplementary Section 3.1.

The United States contributed more than 20% of reported cases globally during the first three outbreaks. Since the end of May 2020, a series of protests against police brutality and racism have been widespread in the US and many regions across the world. Mass gatherings and physical contact during the Floyd protests resulted in a significant increase in contact rates and susceptible supply (Fig. S10). Although the number of protests across the US peaked in early June and steadily declined afterwards, mass gatherings during the protests caused a 52.2% increase in total cases as of 1 September 2020, as shown in Fig. 1d and Supplementary Section 3.2.

Sustained and intensive public health interventions have drastically disrupted almost entire sectors of society. As a result, signs of pandemic fatigue among policymakers and the public have emerged worldwide. For example, steady declines in the government stringency index have been recorded in Brazil and India since May 2020 [10], which almost coincided with upward trends of transmission rates before the significant rise of daily new cases (Figs S12c and S13c). Pandemic fatigue among the public led to demotivated engagement in protection behaviors, which put people at a higher risk of infection. In India, religious celebrations and other social gatherings had been allowed, which pushed reported daily cases to break the world's highest record. However, under sustained public health interventions, the pandemic curves in Brazil and India could have been flattened, as shown in Fig. 1e and f and Supplementary Section 3.3.

The performed statistical analysis and dynamical simulations in this work both indicate multifaceted influences on COVID-19 transmission dynamics. We found limited weekly and seasonal modulations on COVID-19 evolution, while public behaviors and governmental decisions that determine the frequency of mass gatherings were able to cause abrupt shifts in the daily new cases. If gathering activities could be accurately parameterized, then reliable predictions of COVID-19 cases are achievable. Additionally, our study also highlights the decisive role of NPIs. Given the facts of emerging SARS-CoV-2 variants and unguaranteed effectiveness of developed vaccines, NPIs remain one of the most effective measures to control the epidemic in the foreseeable future before high levels of vaccine-mediated protection can be achieved across the world.

## Supplementary Material

nwab100_Supplemental_File
