# Supplementary material for: The oscillation-outbreaks characteristic of the COVID-19 pandemic
Source: Natl Sci Rev. 2021 Jun 8;8(8):nwab100. doi: 10.1093/nsr/nwab100 (PMC8344697; doi:10.1093/nsr/nwab100)
Supplement: nwab100_Supplemental_File [file nwab100_supplemental_file.docx]

**Supplementary Information for**

**The oscillation-outbreaks characteristic of the COVID-19 pandemic**

Jianping Huang (黄建平)^1,^*, Xiaoyue Liu (刘晓岳)^1^, Li Zhang (张立)^1^,

Yingjie Zhao (赵颖洁)^1^, Danfeng Wang (王丹凤)^1^, Jinfeng Gao (高金凤)^1^

Xinbo Lian (连鑫博)^1^, and Chuwei Liu (刘楚薇)^1^

**Affiliations:**

^1^Collaborative Innovation Center for Western Ecological Safety (CIWES), Lanzhou University, Lanzhou 730000, China.

*Correspondence to: Jianping Huang (hjp@lzu.edu.cn).

# The Ensemble Empirical Mode Decomposition

We use the ensemble empirical mode decomposition (EEMD) method to analyze the time series of global pandemic data, which is an adaptive and temporal local data analysis method [1]. The EEMD is a time series analysis method based on the empirical mode decomposition (EMD) method [2]. which decomposes complicated data series into finite quasi-periodic components at different frequencies and is suitable for adaptive analysis of nonlinear and non-stationary time series. The EMD/EEMD method has been used to analyze nonlinear and non-stationary data in the climatic and oceanic analysis [3–4] and for biomedical signal processing [5].

Derived from the EMD method, the EEMD method has various improvements [2]. White noise is added to the original sequence, and the sequence is decomposed into a set of amplitude–frequency-modulated oscillatory components (IMFs, intrinsic mode functions). These steps are repeated using a different white noise sequence each time, and the corresponding intrinsic mode functions are obtained as the final decomposition result. The detailed procedures can be found in previous studies [1–2]. A python module (PyEMD) for EEMD is available at <https://www.github.com/laszukdawid/PyEMD> [6]. Since the prevalence of COVID-19 varies significantly across countries, for comparison we scale the time series of each country so that they range between 0 and 1 before conducting EEMD.

The following figures show the EEMD decomposition result for the daily confirmed and death cases for the Northern Hemisphere (NH, Fig. S1) and the Southern Hemisphere (SH, Fig. S2). The oscillation on the weekly scale is averaged to provide further insight into the weekly cycle (Fig. S3). We also examined the sensitivity of the EEMD components to the different spans of the same data (Figs. S4 and S5). The results showed that time selection has little impact on the oscillatory pattern decomposed by EEMD. This indicates high validity of the weekly and seasonal components in the time-series.


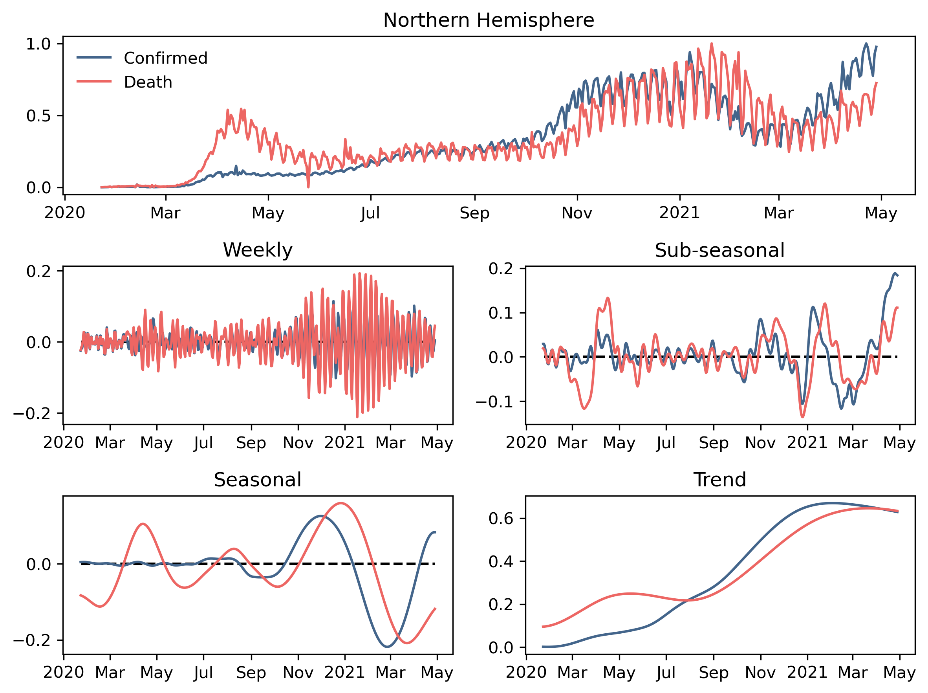


**Figure S1.** EEMD analysis of the time-series (daily confirmed and daily death cases) in the Northern Hemisphere.


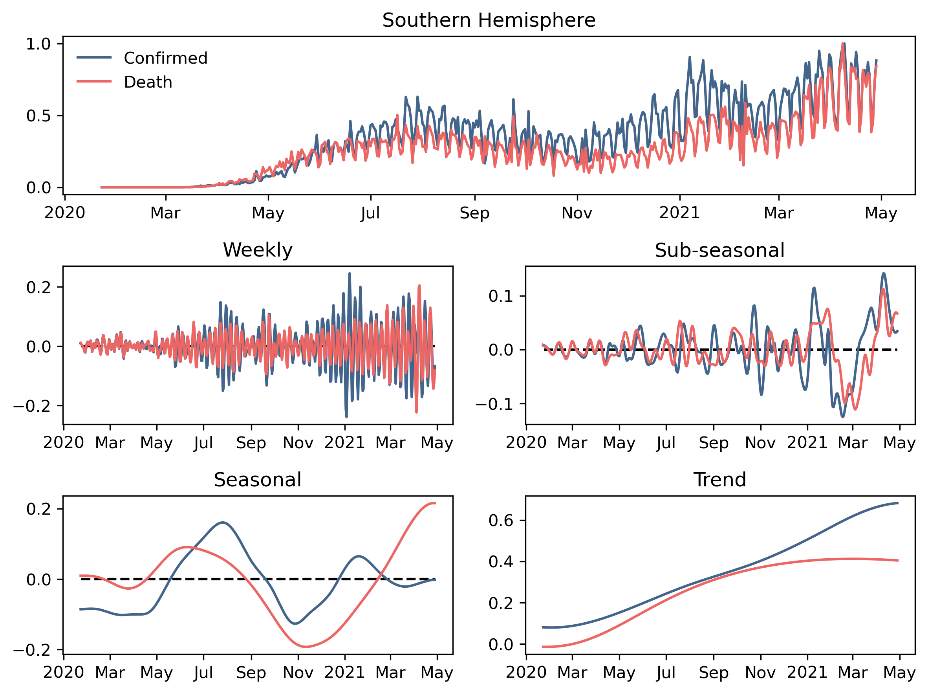


**Figure S2.** Same as Fig.S1, but for the Southern Hemisphere.


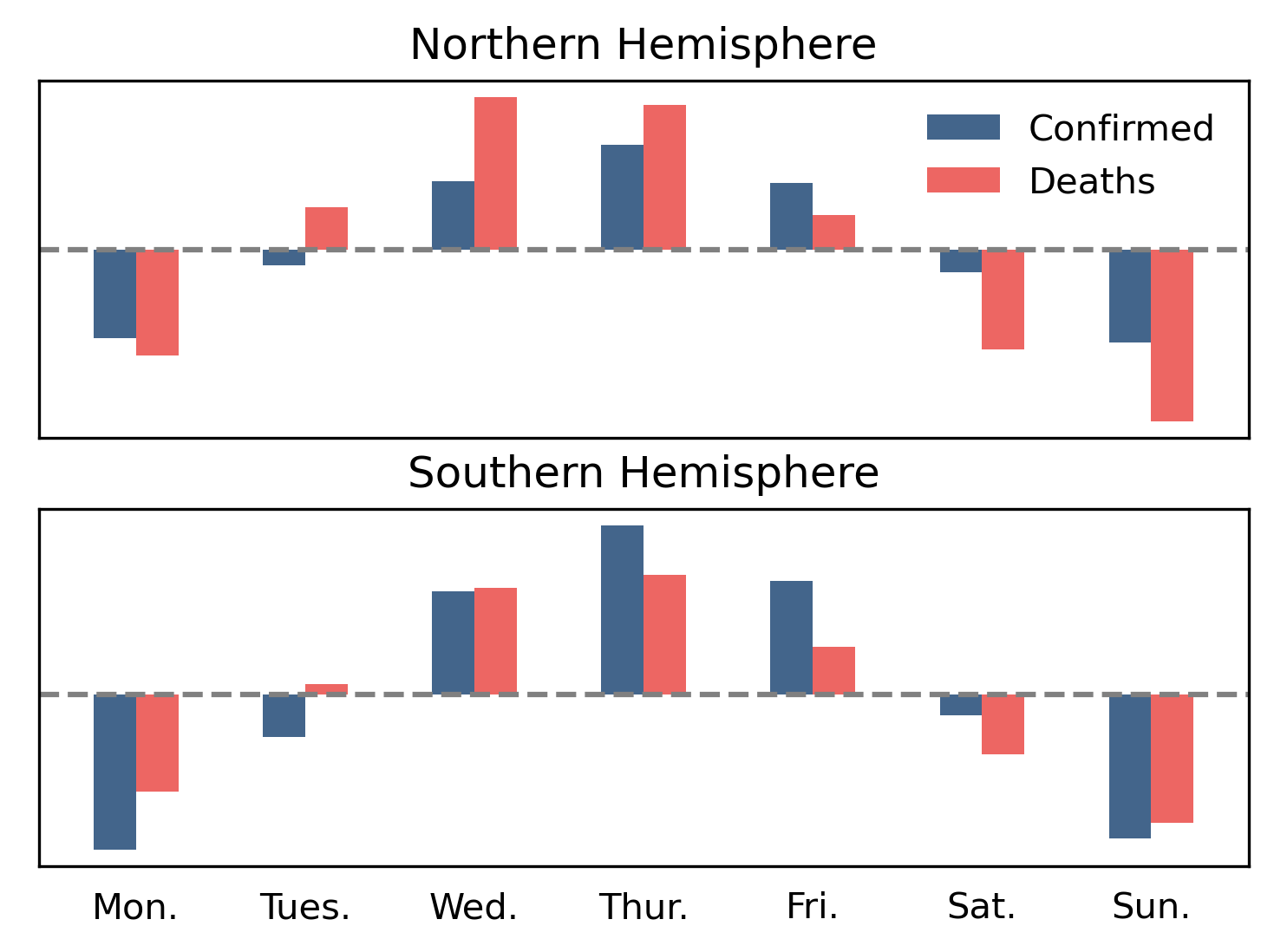


**Figure S3.** Weekly oscillation of daily confirmed and death cases derived by EEMD method.


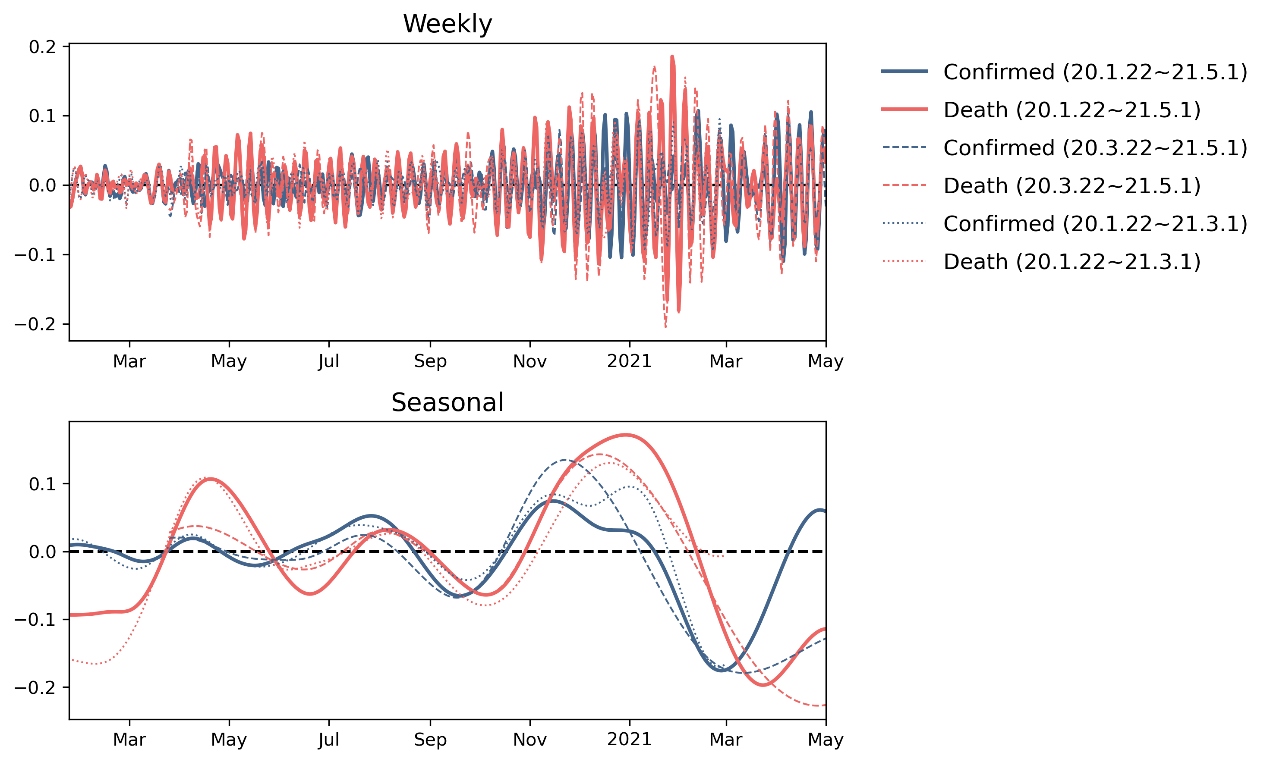


**Figure S4.** Oscillation components of daily confirmed and death cases of NH in different time periods selected. The periods 20.1.22 –21.5.1, 20.3.22 –21.5.1, and 20.1.22 –21.3.1 are examined here.


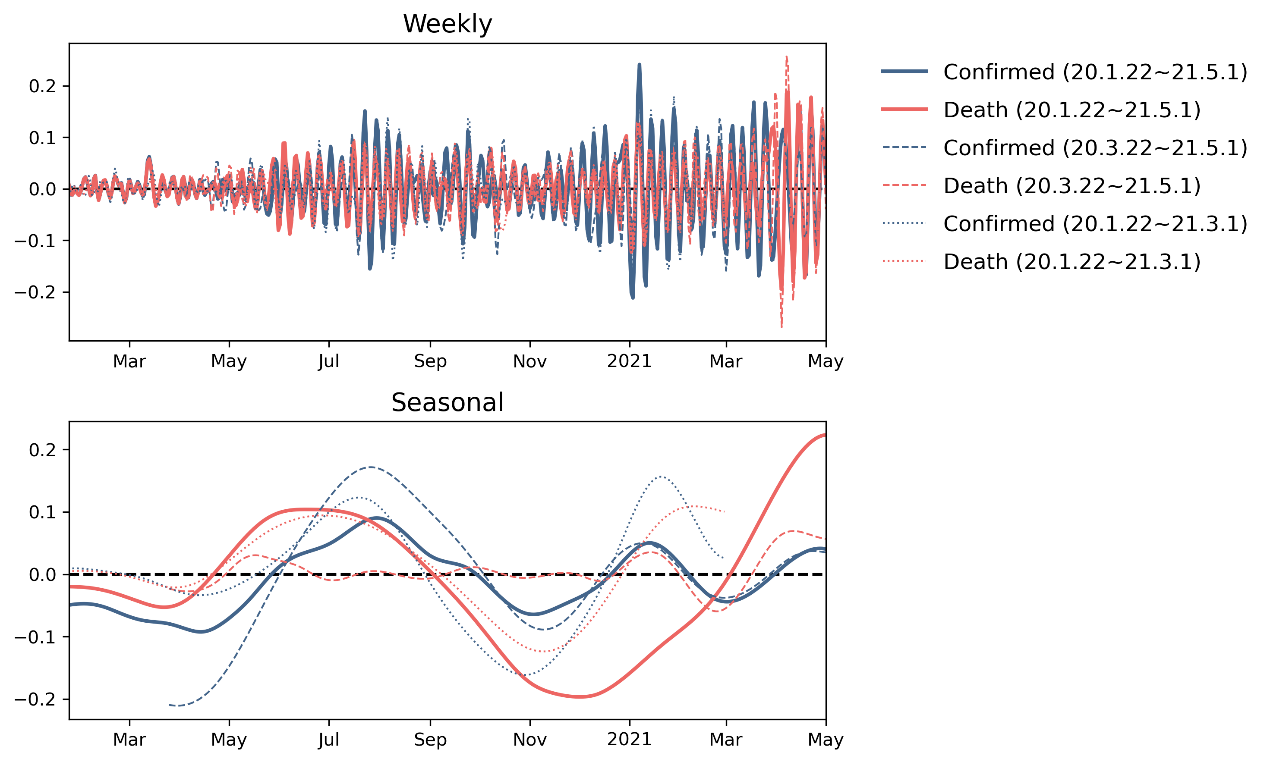


**Figure S5.** Same as Fig. S4, but for SH.

# Anomaly detection

An anomaly detection algorithm is applied to identify significant level shifts in a time series. The algorithm slides two time-windows side-by-side and keeps tracking the difference between their median values. Whenever the statistics in the left and right windows are significantly different, it indicates an abrupt change around this time point. This algorithm requires that both windows should be long enough to capture stable status. In this work, we set window size 30 days, and identifies time points as anomalous shifts when the absolute difference in median values between the two windows are beyond 62.5% quantile. The upper panel of Fig. S6 shows the time-series of global daily cases, and the lower panel shows the difference of the medians between left and right windows, with the red lines indicating the values of 60% quantile. Based on this method, the following points are identified: 2020.03.17, 2020.03.28, 2020.06.11, 2020.07.17, 2020.09.27, 2020.11.20, 2021.01.09, 2021.02.16, 2021.03.02. These points divide the time-series into 10 segments. For the convivence of analysis, the segments (2021.02.16, 2021.03.02) are merged with the neighboring segments. Finally, four major rapid growing periods of global daily new cases are identified:

- 2020.03.17~2020.03.28
- 2020.06.11~2020.07.17
- 2020.09.27~2020.11.20
- 2021.02.16~


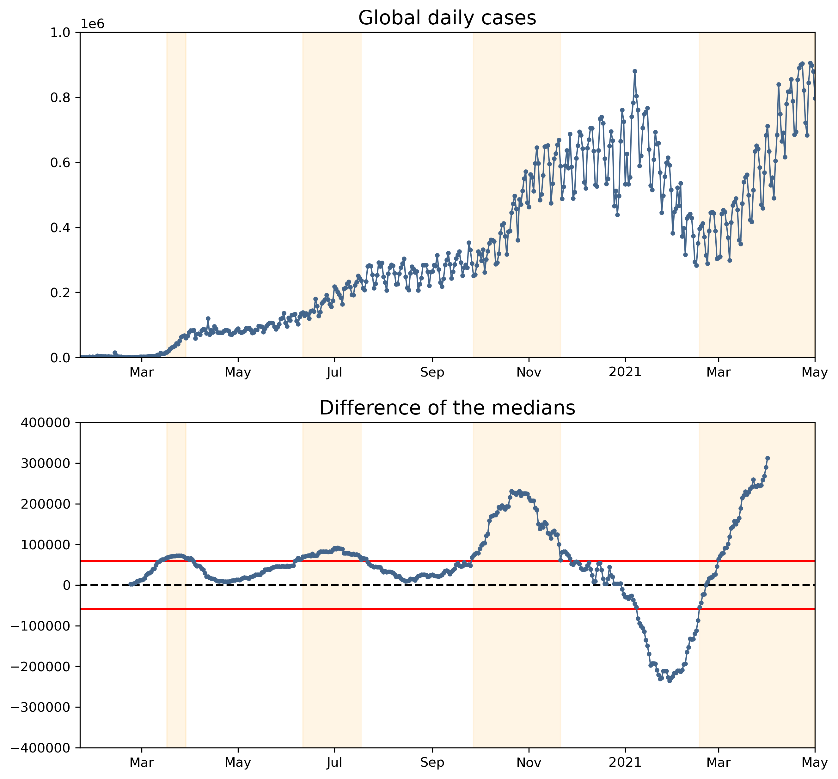


**Figure S6.** The segmentation of COVID-19 daily cases based on an anomaly detection algorithm. The red line in the lower panel is the threshold (60% quantile of the median difference) to detect the staged change. The periods with the values higher (lower) than the red line are identified as the rapid growing periods, illustrating by the vertical dashed line.


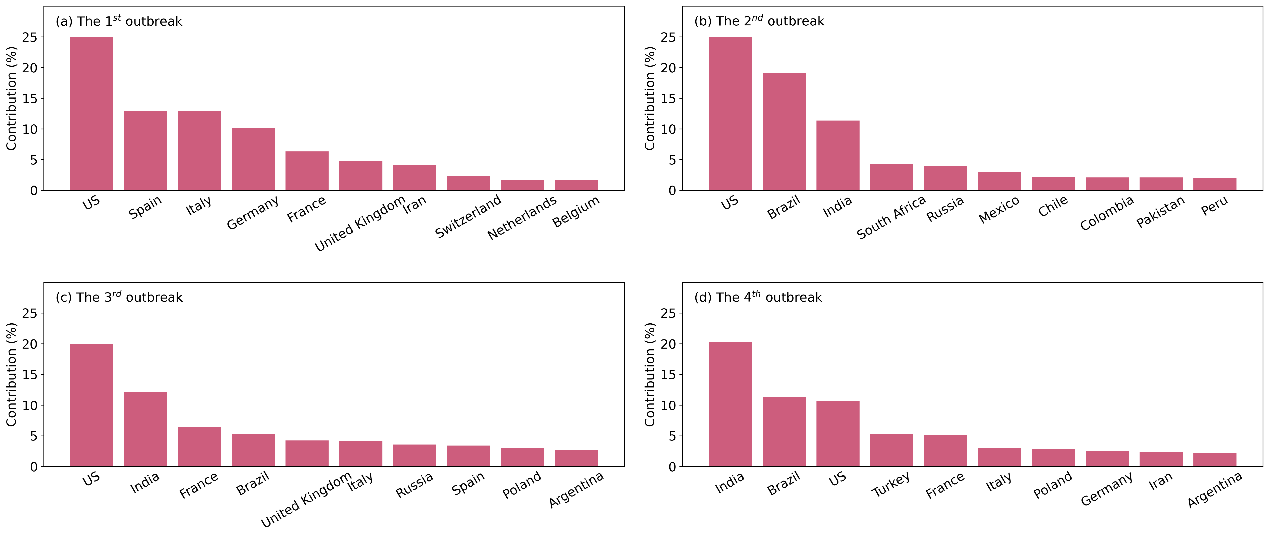


**Figure S7**. The contribution of the top-10 countries (regions) to the growth in the first (a), second (b), third (c) and fourth (d) outbreaks, respectively.

# The modified SEIR model

The SEIR model defines seven states of the disease: susceptible cases (S), protected cases (P), potentially infected cases (E, infected cases in a latent period), infectious cases (I, infected cases that have not been quarantined), quarantined cases (Q, confirmed and quarantined cases), recovered cases (R), and cases of mortality (D). The sum of the six categories is equal to the total population (N) at any time.

S + P + E + I + Q + R + D = N

The modified SEIR model is able to emulate the time curve of an outbreak [7]. The model consists of the following equations:

| $\frac{dS\left( t \right)}{dt}=-\frac{\beta I\left( t \right)S\left( t \right)}{N}-\alpha S\left( t \right)$ | (1) |
| --- | --- |
| $\frac{dP\left( t \right)}{dt}=\alpha S\left( t \right)$ | (2) |
| $\frac{dE\left( t \right)}{dt}=\frac{\beta I\left( t \right)S\left( t \right)}{N}-\gamma E\left( t \right)$ | (3) |
| $\frac{dI\left( t \right)}{dt}=\gamma E\left( t \right)-\delta I\left( t \right)$ | (4) |
| $\frac{dQ\left( t \right)}{dt}=\delta I\left( t \right)-\lambda Q\left( t \right)-\kappa Q\left( t \right)$ | (5) |
| $\frac{dR\left( t \right)}{dt}=\lambda Q\left( t \right)$ | (6) |
| $\frac{dD\left( t \right)}{dt}=\kappa Q \left( t \right)$ | (7) |

The parameters are optimized based on the data of confirmed (Q), recovered (R), and death cases (D) from COVID-19 Data Repository by the Center for Systems Science and Engineering (CSSE) at Johns Hopkins University [8] using the nonlinear least-squares algorithm (Levenberg-Marquardt method). The values of parameters ($\alpha, \beta_{0}, \beta_{1}, \gamma,\lambda, \delta,$ and $\kappa$) are determined when the residual reaches its minimum. To define a realistic range of these parameters and understand their meaning, the upper and lower boundaries of the parameters are set (see Table S1).

**Table S1.** Parameters in the modified SEIR model

| **Parameters** | **Meaning** | **Range** |
| --- | --- | --- |
| $\alpha$ | protection rate | -1~1* |
| $\beta$ | transmission rate | 0~5 |
| $\gamma$ | reciprocal of latent time | 0~1 |
| $\delta$ | reciprocal of the delay time to transit the population from compartment I to Q | 0~1 |
| $\lambda$ | recovery rate | 0~1 |
| $\kappa$ | death rate | 0~1 |
| *The protection rate ($\alpha$) can be negative when measures of social distancing are relaxed. When $\alpha<0$, the population in compartment P (the protected) can return to compartment S (the susceptible) and becomes vulnerable to the virus again. | | |

Since the local authorities would adjust the intervention strategy in different phases of the outbreaks, the parameters in the model should variate accordingly. In addition, the parameter describing the transmission capacity ($\beta$) is not a biological constant, and would also be affected by environmental conditions and behaviors of the infected individuals. Therefore, a rolling window method is employed to retrieve the parameters on a regular basis (window size = 30 days, interval = 5 days, see Fig. S8). The differential equations are numerically solved by the 4^th^ order Runge–Kutta method.


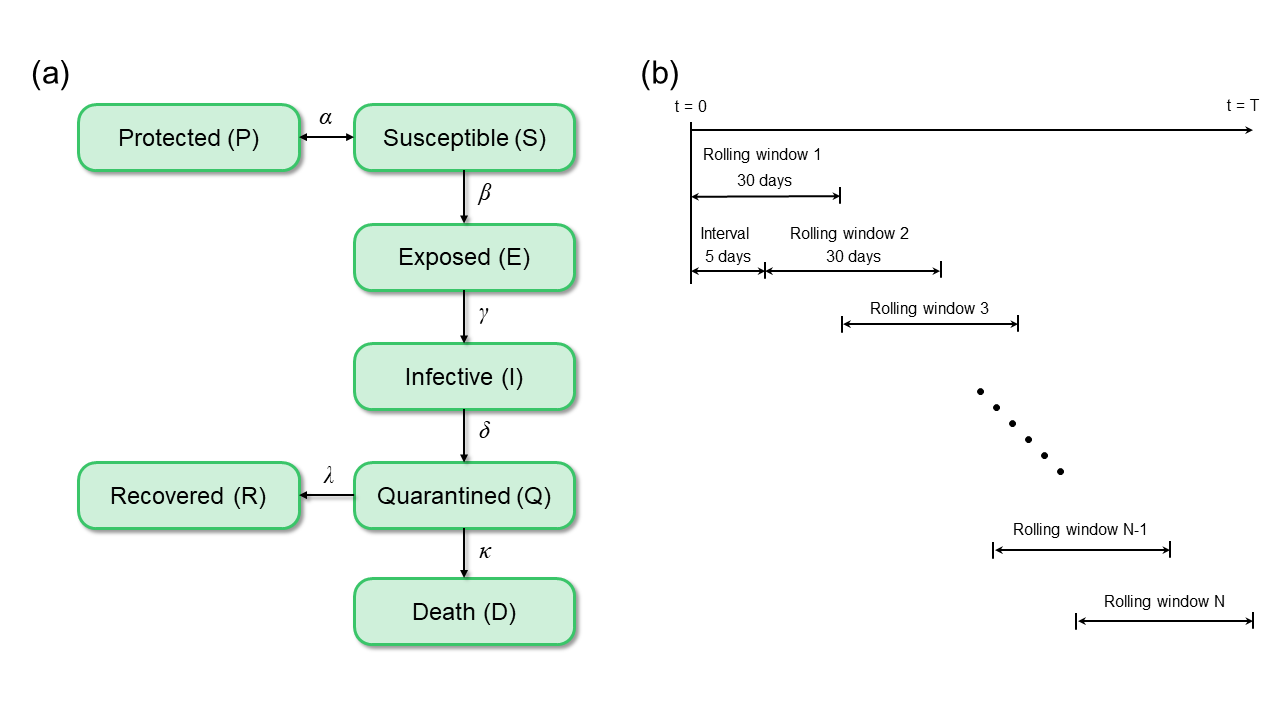


**Figure S8.** The modified SEIR model (a) and rolling window method (b) (reproduced from Liu et al., 2021[9]).

The reported data in the selected countries are preprocessed before they are introduced to fit the parameters. In the initial stage of the outbreak, the quality of the reported data might be influenced by the lack of local medical capacity. The introduction of these early-stage data could result in large uncertainty of the model simulation. Therefore, we set an empirical threshold to introduce the reported data into our model when the local confirmed cases reach 1,000. The time range of the data introduced in the simulation for each country is listed in Table S2.

**Table S2.** Time range of data input for simulation in each country

| **Country** | **Start date** | **Confirmed cases on the start date** |
| --- | --- | --- |
| United States | 2020-3-11 | 1147 |
| India | 2020-3-29 | 1024 |
| Brazil | 2020-3-21 | 1021 |
| Russia | 2020-3-27 | 1036 |

## Simulations for Russia: case importation


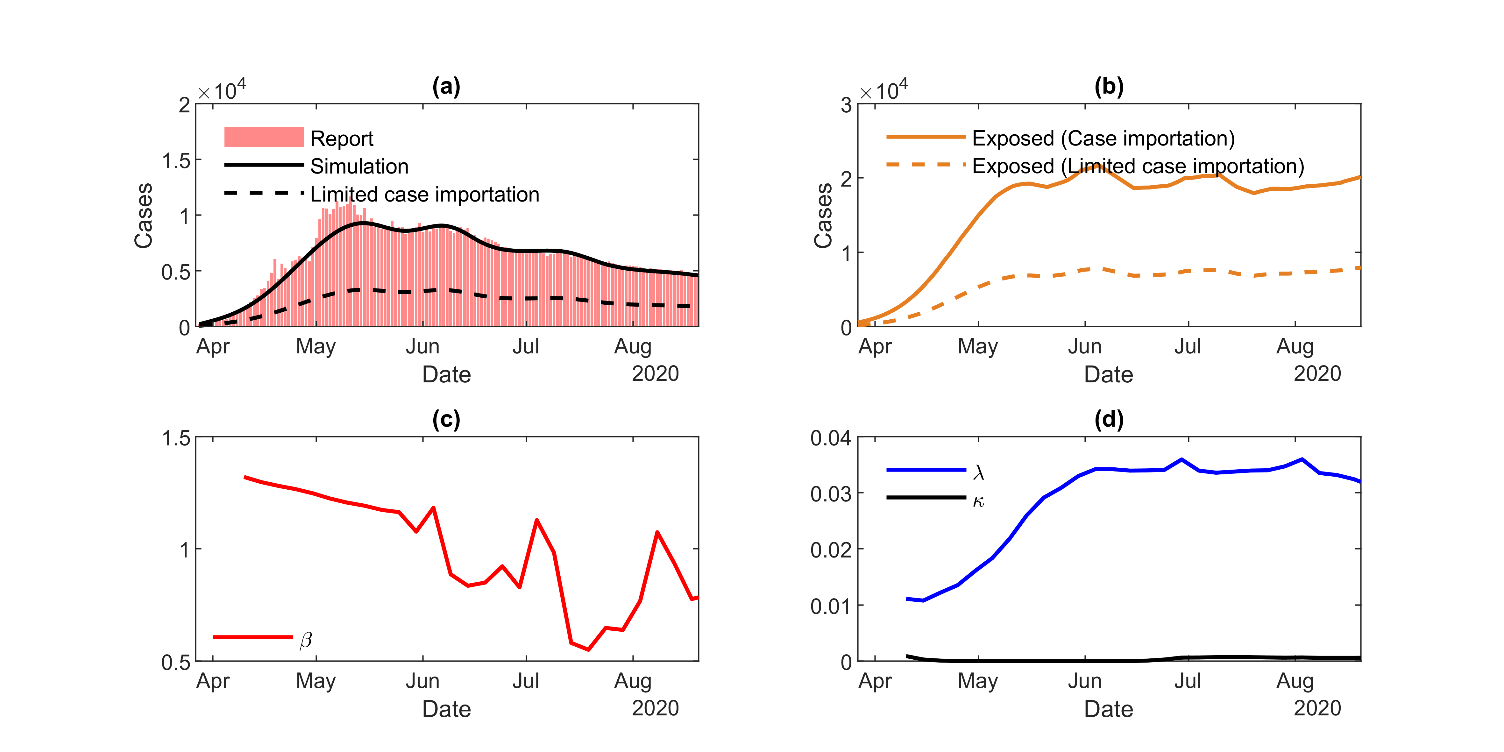


**Figure S9.** Simulations for Russia. (a) reported and simulated daily cases. (b) simulations of the Exposed cases (Group E). The solid back line in (a) shows the simulated daily new cases, while the dotted line shows the simulated daily new cases in scenario of limited case importation. The red line in (c) denotes the transmission rate ($\beta$). The blue and black lines in (d) denote the recovery and death rate ($\lambda$ and $\kappa$), respectively.

For Russia, the flight data (see the bar in Fig. 1b) did not show a significant decline of inbound flights from other European countries until mid-March, 2020. The delayed entrance management policies would substantially contribute to the early introduction of COVID-19 cases from Europe to Russia. The flight data could be freely accessible at <https://doi.org/10.5281/zenodo.3931948> [10]. This database presents a comprehensive air traffic dataset, derived and enriched from the full OpenSky data. It spans all flights seen by the network's more than 3500 members between 1 January 2019 and 1 July 2020. The archive is being updated every month and for the first 18 months includes 41 900 660 flights, from 160 737 aircraft, which were seen to frequent 13 934 airports in 127 countries.

Since most of the reported cases during this period are related to imported cases [11], lower number of undetected cases at the initial stage (I_0_) is associated with lower number of imported cases. Therefore, the value of I_0_ is a key indicator that reflects the effectiveness of NPIs that are related to entrance management.

Within the dynamic framework of our model, when the other parameters ($\beta$, $\lambda,$ and $\kappa$, etc.) in the model remain unchanged, reducing the value of I_0_ in a simulation would produce a scenario of early entrance management. To isolate the impact of case importation in the early stage of the outbreak in Russia, the parameters ($\beta$, $\lambda$ and $\kappa$, etc.) in the model were first determined based on the observed data since 2020-3-21, and the data on 2020-3-21 is the initial condition that drives the model. We then run the model again with the same parameters retrieved, but replace the original initial condition with the reported data on 2020-3-16 (5 days ahead). The simulation output (dashed line in Figs. S9a & b) could be interpreted as the scenario where the same policies were implemented 5 days earlier.

## Simulations for US: nation-wide protests


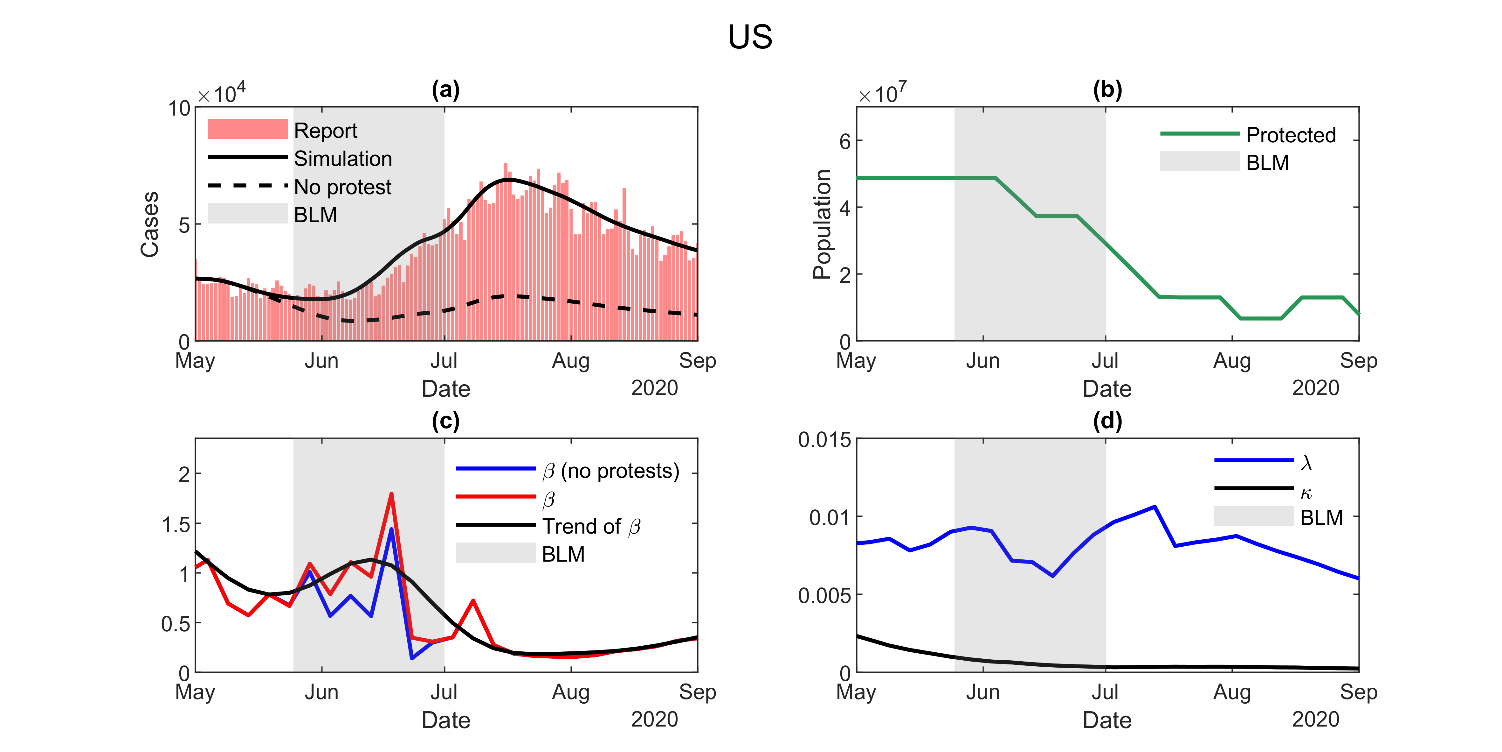


**Figure S10.** Simulations for US. (a) reported and simulated daily cases. (b) simulations of the Protected cases (groups P). The solid back line in (a) shows the simulated daily new cases, while the dotted line shows the simulated daily new cases in scenario of limited case importation. The red and blue lines in (c) denote the transmission rates ($\beta$) in two different scenarios, and the black line in (c) denote the trend of transmission rate. The blue and black lines in (d) denote the recovery and death rate ($\lambda$ and $\kappa$), respectively.

We first use the rolling window method to retrieve the time-varying model parameters, and then we compare these parameters with the timeline of the nationwide Floyd protests. The data of protests across the world could be freely accessible via <https://acleddata.com/>. This website provides the collections of real-time data on the locations, dates, actors, fatalities, and types of all reported political violence and protests events across Africa, the Middle East, Latin America & the Caribbean, East Asia, South Asia, Southeast Asia, Central Asia & the Caucasus, Europe, and the United States of America. We focus on the protests in the US. During our research period, the protests across the US mainly focus on the following two issues: COVID-19 lockdowns and the death of George Floyd.

Our simulations indicate a significant upward trend of transmission rate ($\beta$) during the BLM (Black Lives Matter) protests (grey shadings), and a decline in the protected populations. Mass gatherings, including protests, would lead to increased contact frequency among people, and elevate the risk of infection. To isolate the impact of protests on the increasing transmission rate, we used the EEMD method to decompose the time-series of $\beta$, and subtracted the components related to the demonstrations from the original time-series. The modified time-series of $\beta$ was (blue line in Fig. S10c) then put back to the model to run another simulation to produce the scenario without the impact of protests.

## Simulations for Brazil and India: pandemic fatigue


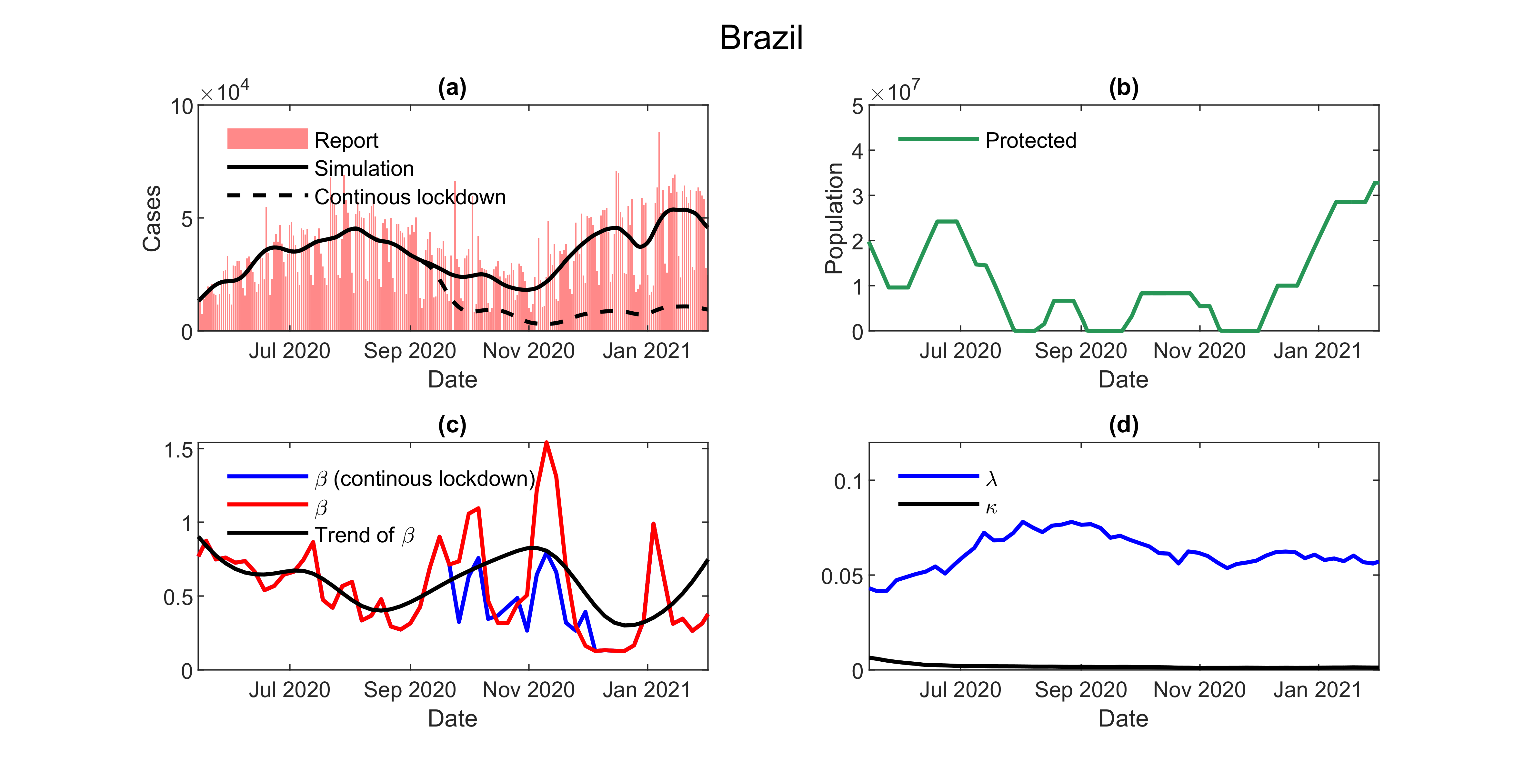


**Figure S12.** Same as Figure S11, but for Brazil.

The outbreaks in Brazil and India are also reproduced based on the modified SEIR model and the rolling window method. The trends of the protected population in the simulations of Brazil and India are basically consistent with the trends of the stringency index in these two countries. And upward trends of transmission rates ($\beta$) in both countries are also associated with the decline in the stringency index. This indicates that the outbreaks can be faithfully reproduced within the dynamic framework of the model.

The scenarios of continuous lockdown are simulated in a similar way. The significant upward trends of $\beta$ are replaced by the stable components of $\beta$ time-series decomposed via the EEMD method. The modified time-series of $\beta$ is (blue line in Figs. S12c and S13c) then put back to the model to run another simulation to produce the scenario without the impact of protests.


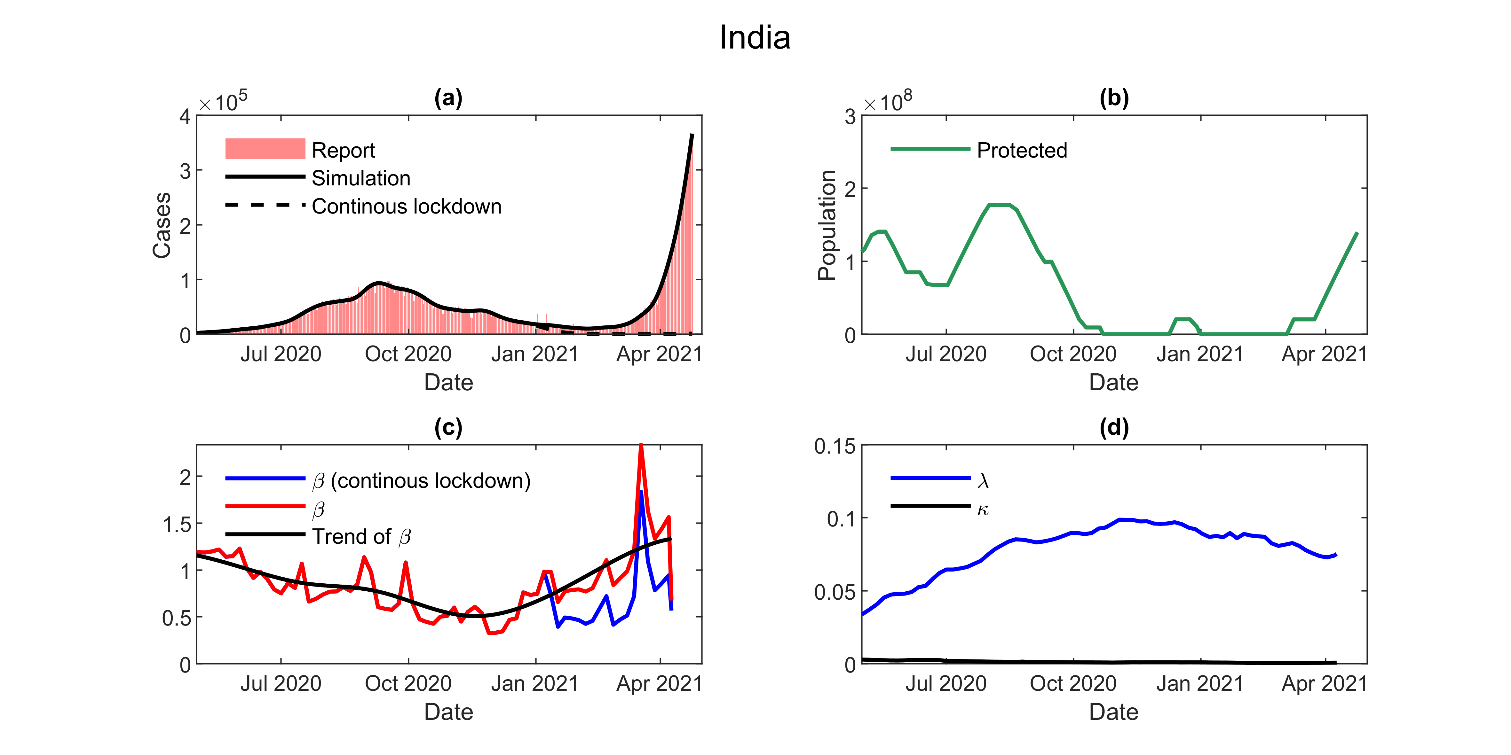


**Figure S13.** Same as Figure S11, but for India.

# Reference

1. Z. WU & N. E. HUANG, ENSEMBLE EMPIRICAL MODE DECOMPOSITION: A NOISE-ASSISTED DATA ANALYSIS METHOD. *Advances in Adaptive Data Analysis*, **01** (2009) 1–41. https://doi.org/10.1142/S1793536909000047.

2. N. E. Huang & Z. Wu, A review on Hilbert-Huang transform: Method and its applications to geophysical studies. *Reviews of Geophysics*, **46** (2008) RG2006. https://doi.org/10.1029/2007RG000228.

3. F. Ji, Z. Wu, J. Huang, & E. P. Chassignet, Evolution of land surface air temperature trend. *Nature Climate Change*, **4** (2014) 462–466. https://doi.org/10.1038/nclimate2223.

4. X. Chen, X. Zhang, J. A. Church, C. S. Watson, M. A. King, D. Monselesan, B. Legresy, & C. Harig, The increasing rate of global mean sea-level rise during 1993–2014. *Nature Climate Change*, **7** (2017) 492–495. https://doi.org/10.1038/nclimate3325.

5. M. A. Colominas, G. Schlotthauer, & M. E. Torres, Improved complete ensemble EMD: A suitable tool for biomedical signal processing. *Biomedical Signal Processing and Control*, **14** (2014) 19–29. https://doi.org/10.1016/j.bspc.2014.06.009.

6. D. Laszuk, Python implementation of Empirical Mode Decomposition algorithm. *GitHub Repository*, (2017).

7. A. Godio, F. Pace, & A. Vergnano, SEIR Modeling of the Italian Epidemic of SARS-CoV-2 Using Computational Swarm Intelligence. *International Journal of Environmental Research and Public Health*, **17** (2020) 3535. https://doi.org/10.3390/ijerph17103535.

8. E. Dong, H. Du, & L. Gardner, An interactive web-based dashboard to track COVID-19 in real time. *The Lancet Infectious Diseases*, **20** (2020) 533–534. https://doi.org/10.1016/S1473-3099(20)30120-1.

9. X. Liu, J. Huang, C. Li, Y. Zhao, D. Wang, Z. Huang, & K. Yang, The role of seasonality in the spread of COVID-19 pandemic. *Environmental Research*, **195** (2021) 110874. https://doi.org/10.1016/j.envres.2021.110874.

10. M. Strohmeier, X. Olive, J. Lübbe, M. Schäfer, & V. Lenders, Crowdsourced air traffic data from the OpenSky Network 2019–2020. *Earth System Science Data*, **13** (2021) 357–366. https://doi.org/10.5194/essd-13-357-2021.

11. A. B. Komissarov, K. R. Safina, S. K. Garushyants, A. V. Fadeev, M. V. Sergeeva, A. A. Ivanova, D. M. Danilenko, D. Lioznov, O. V. Shneider, N. Shvyrev, V. Spirin, D. Glyzin, V. Shchur, & G. A. Bazykin, Genomic epidemiology of the early stages of the SARS-CoV-2 outbreak in Russia. *Nature Communications*, **12** (2021) 649. https://doi.org/10.1038/s41467-020-20880-z.
